# Antiviral Resistance and Phage Counter Adaptation to Antibiotic-Resistant Extraintestinal Pathogenic *Escherichia coli*

**DOI:** 10.1128/mBio.00211-21

**Published:** 2021-04-27

**Authors:** Keiko C. Salazar, Li Ma, Sabrina I. Green, Jacob J. Zulk, Barbara W. Trautner, Robert F. Ramig, Justin R. Clark, Austen L. Terwilliger, Anthony W. Maresso

**Affiliations:** a Department of Integrative Molecular and Biomedical Science, Baylor College of Medicine, Houston, Texas, USA; b Department of Molecular Virology and Microbiology, Baylor College of Medicine, Houston, Texas, USA; c School of Biological and Physical Sciences, Northwestern State University, Natchitoches, Louisiana, USA; d Michael E. DeBakey Veterans Affairs Medical Center, Houston, Texas, USA; e Department of Medicine, Baylor College of Medicine, Houston, Texas, USA; Universidade de Sao Paulo

**Keywords:** antibiotic resistance, bacterial evolution, bacteriophage evolution, bacteriophage resistance, bacteriophage therapy, host-pathogen interactions, multidrug resistance, sepsis

## Abstract

Extraintestinal pathogenic Escherichia coli (ExPEC), often multidrug resistant (MDR), is a leading cause of urinary tract and systemic infections. The crisis of emergent MDR pathogens has led some to propose bacteriophages as a therapeutic. However, bacterial resistance to phage is a concerning issue that threatens to undermine phage therapy. Here, we demonstrate that E. coli sequence type 131, a circulating pandemic strain of ExPEC, rapidly develops resistance to a well-studied and therapeutically active phage (ϕHP3). Whole-genome sequencing of the resisters revealed truncations in genes involved in lipopolysaccharide (LPS) biosynthesis, the outer membrane transporter *ompA*, or both, implicating them as phage receptors. We found ExPEC resistance to phage is associated with a loss of fitness in host microenvironments and attenuation in a murine model of systemic infection. Furthermore, we constructed a novel phage-bacterium bioreactor to generate an evolved phage isolate with restored infectivity to all LPS-truncated ExPEC resisters. This study suggests that although the resistance of pandemic E. coli to phage is frequent, it is associated with attenuation of virulence and susceptibility to new phage variants that arise by directed evolution.

## Abstract (precis)

Extraintestinal pathogenic Escherichia coli (ExPEC), often multidrug resistant (MDR), is a leading cause of urinary tract and systemic infections. The crisis of emergent MDR pathogens has led some to propose bacteriophages as a therapeutic.

## INTRODUCTION

Multidrug-resistant (MDR) infections are a major global concern, resulting in significant morbidity and mortality. In the United States alone >2.8 million MDR infections occur annually, 35,900 of which result in death (1, 2). Those who survive their infections often suffer chronic cases lasting months or even years (3). Exacerbating this crisis, the pipeline for antibiotic development is slow, and resistant strains rapidly develop in the wake of new drugs (4). The family *Enterobacteriaceae* provides an urgent threat of MDR infections, due in part to strains of Escherichia coli (2). Extraintestinal pathogenic E. coli (ExPEC), a pathotype of the larger E. coli superfamily, is a natural inhabitant of the human gastrointestinal microbiome. ExPEC strains are unique in their ability to translocate and cause subsequent infections in immunocompromised individuals (5, 6). When they do, they cause an array of serious illnesses, including urinary tract infections (UTIs), bacteremia, sepsis, and neonatal meningitis (6).

A promising response to MDR infections is bacteriophage (phage) therapy. Viruses which infect bacteria, phages are environmentally ubiquitous, host specific, and effective at infecting MDR bacterial strains (7–11). Importantly, they have been shown to be safe and effective in animal and compassionate-use human trials (3, 7–15). Phage use the replication machinery of their bacterial host, meaning phage mutation rates are directly influenced by those of that host (16); as such, phages may rapidly adapt to target strains of bacteria. However, due to the cognate rates of evolution between a phage and its host, a mixed population of phages and bacteria will result in an evolutionary arms race (17–23). Consequently, phage-resistant bacteria are likely to develop. Interestingly, studies have found that in some instances, bacteria which develop resistance to phage lose other fitness advantages in exchange (9, 24–29). Finally, though phage-bacterial coevolution is well studied *in vitro*, it is poorly understood under *in vivo* conditions. This leaves many questions as to mechanisms of resistance that may develop during treatment, the state of the pathogen after it has undergone these changes, and whether new phage can be reliably isolated or evolved that counter such changes.

Here, we explore the phage-bacterial arms race by examining adaptations which facilitate bacterial resistance and subsequent phage reinfection. We found that key changes at the phage binding site drives the outcome of this interaction for pathogenic E. coli and a previously used therapeutic phage, ϕHP3. Strikingly, not only does phage pressure drive a successive loss of fitness in human blood and decreased virulence overall but also phages utilized subtle counteradaptations that expanded their host range to resistors when developed by a novel directed-evolution bioreactor.

## RESULTS

### Selection of phage-resistant bacterial isolates.

**FIG 1:**
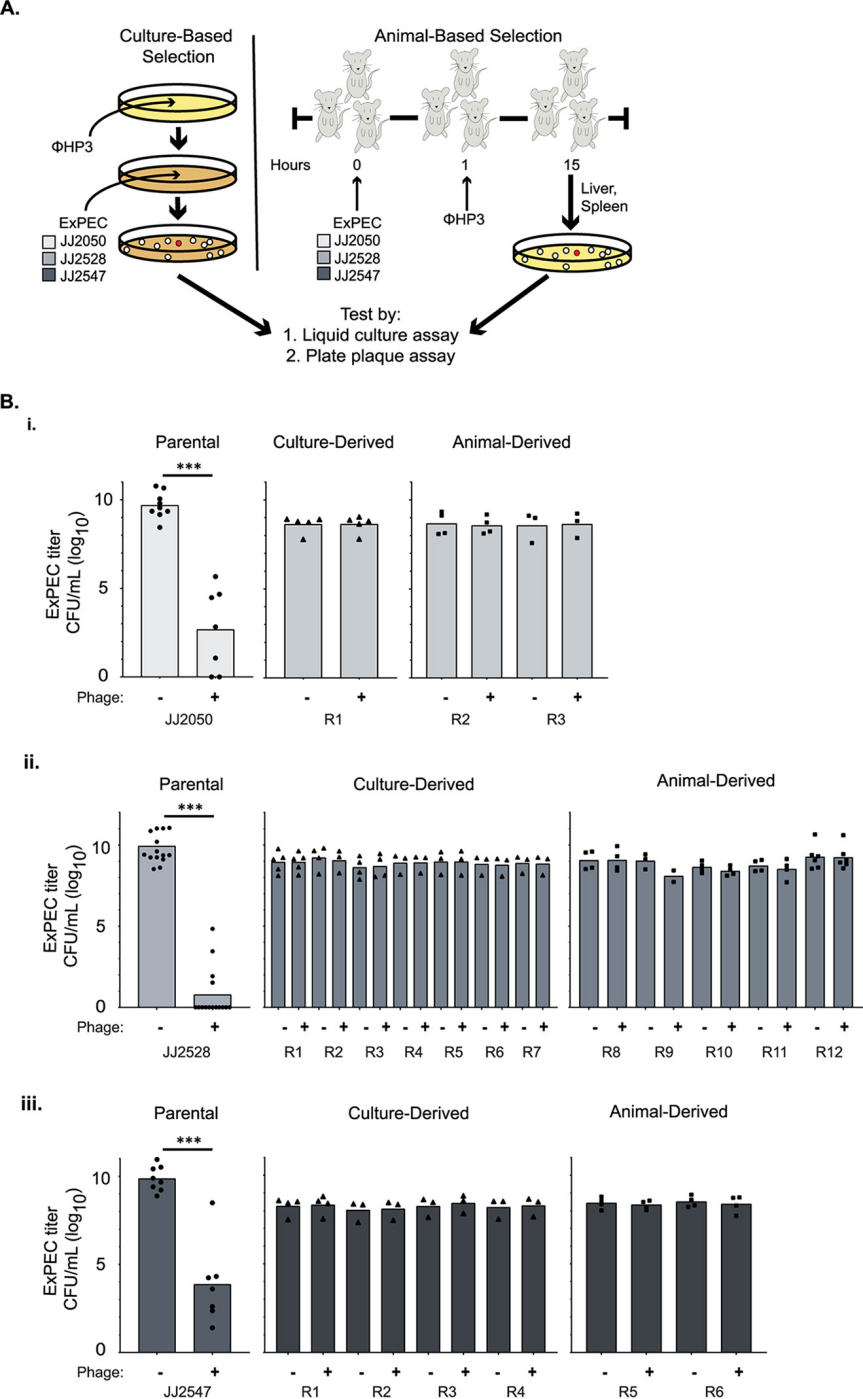
Isolation of phage-resistant ExPEC. Three clinical ExPEC isolates—JJ2050, JJ2528, and JJ2547—were used to develop resisters against ϕHP3. (A) Resisters were isolated using either a culture- or murine-based method. In both cases, parental strains were subjected to selective pressure in the presence of ϕHP3. (B) Isolate titers were determined in liquid culture after 4.5 h of incubation in LB, with or without the presence of ϕHP3. (B) Panel B is grouped by parental isolate: JJ2050 (i), JJ2528 (ii), and JJ2547 (iii). *P* values were determined by Student *t* test or Mann-Whitney test, where necessary. *, *P* < 0.05; **, *P* < 0.01; ***, *P* < 0.01. Bars represent the average titers. Each data point represents the average of three parallel technical replicates.

ϕHP3 is an extensively characterized, lytic phage that is an effective therapeutic in a murine sepsis model (8, 30, 31). It has also, with single-patient INDs (investigational new drugs; approved by the U.S. Food and Drug Administration for compassionate use), been successfully used in two patients with E. coli infections (15). Three clinically derived ExPEC strains—JJ2050, JJ2528, and JJ2547—were selected as MDR pathogens capable of causing illness in this model (8, 31). These strains are all of sequence type 131 (ST131) and were isolated from different patients (see Fig. S1 in the supplemental material). When treating mice for ExPEC-derived sepsis, we found that although phage reduced bacterial burden in most animals, some subjects maintained relatively high bacterial levels (8). We hypothesized that, in these cases, phage-resistant isolates (“resisters”) had arisen during treatment. To test this hypothesis, we isolated phage resisters by challenging them with ϕHP3 using two different methods (Fig. 1A). The first method, culture-based selection, consisted of streaking an overnight bacterial culture on a Luria broth (LB) plate coated with phage. The second, animal-based selection used our murine sepsis model to recover resisters from phage-treated animals (8). Briefly, mice were given an intraperitoneal (i.p.) injection of each ST131 strain, followed by i.p. injection of phage 1 h later. Bacterial isolates were then recovered from the livers and spleens of the euthanized mice the next day.

10.1128/mBio.00211-21.1 FIG S1 Phylogenetic tree of ST131. The three experimental ST131 strains are shown with their relation to other ST131s and laboratory strain K-12. The numbers on each branch indicate the number of substitutions per site. Download FIG S1, TIF file, 0.2 MB. Copyright © 2021 Salazar et al. 2021 Salazar et al. https://creativecommons.org/licenses/by/4.0/ This content is distributed under the terms of the Creative Commons Attribution 4.0 International license.

Isolates from both strategies were tested for phage resistance by two methods: (i) coincubation in liquid culture and (ii) phage spot assay. For the coincubation assay, isolates were grown for 4.5 h in LB with or without phage. The three parental ST131 strains were readily killed by ϕHP3 (Fig. 1Bi, ii, and iii). Isolates that survived the coincubation selection, however, were refractory to any killing by phage (Fig. 1Bi, ii, and iii). Further, parental isolates yielded a high phage titer by spot assay, but the resisters produced no observable phage progeny (see Fig. S2). These findings indicate the isolates have acquired full resistance to ϕHP3. Between the two isolation strategies, a total of 21 independent resisters were isolated (summarized in Table 1). The majority of resisters had smaller colonies compared to their wild-type (WT) progenitors (see Fig. S3).

**TABLE 1:**
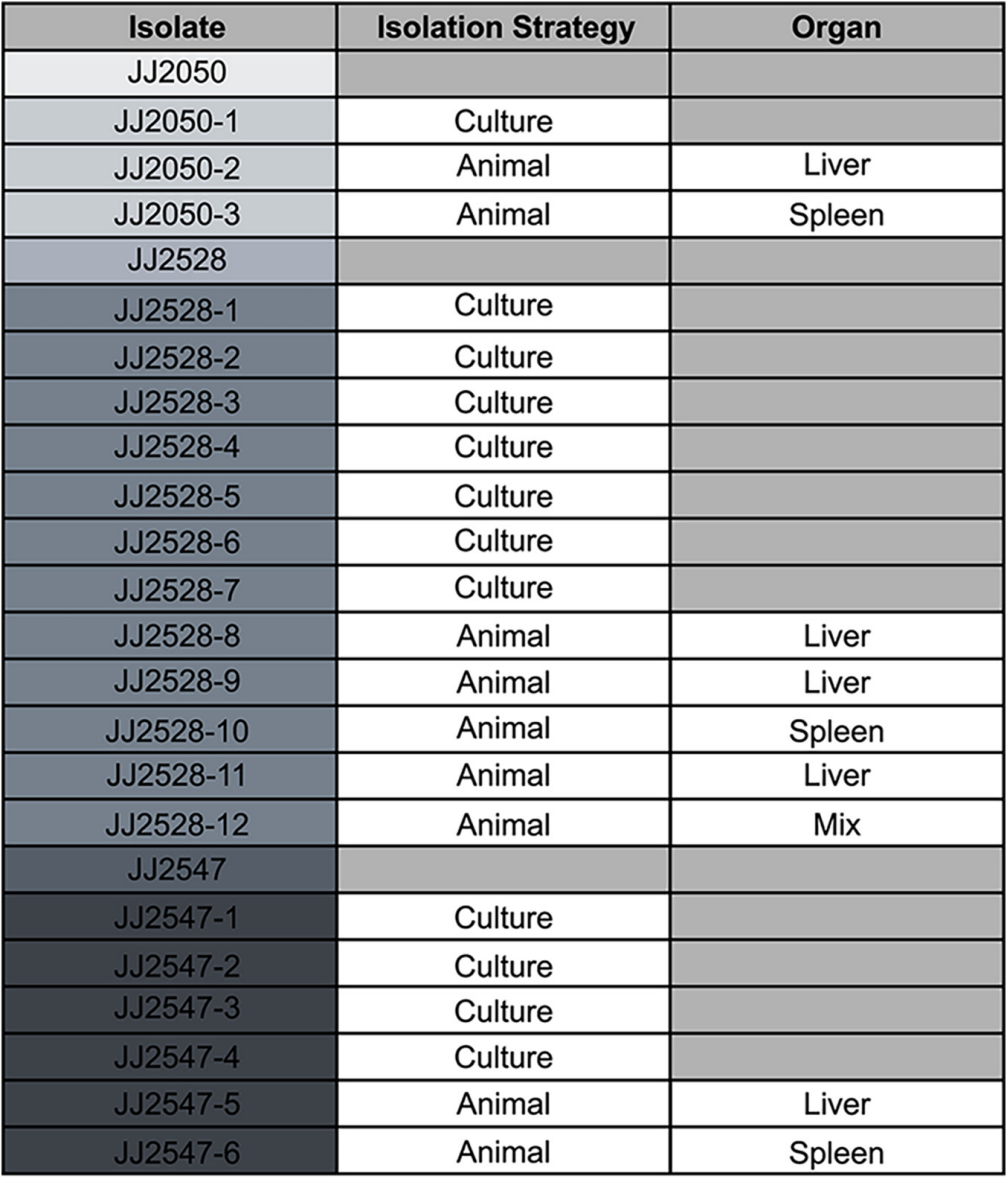
Phage-resistant isolates^*a*^

a A total of 21 combined ϕHP3-resistant isolates were collected from the three parental isolates and two selection methods. Each isolate and its source is listed.

10.1128/mBio.00211-21.2 FIG S2 Isolation of phage-resistant ExPEC. ϕHP3 titer was determined for each isolate using the standard phage spot assay. Panels are grouped by parental isolate: JJ2050 (i), JJ2528 (ii), and JJ2547 (ii). Each data point represents the average of three parallel technical replicates. Download FIG S2, TIF file, 0.4 MB. Copyright © 2021 Salazar et al. 2021 Salazar et al. https://creativecommons.org/licenses/by/4.0/ This content is distributed under the terms of the Creative Commons Attribution 4.0 International license.

10.1128/mBio.00211-21.3 FIG S3 Colonies of phage-resisters. *waaC*- and *hldE*-truncated resisters show smaller colonies than their parental and *ompA*-truncated counterparts. Download FIG S3, TIF file, 0.5 MB. Copyright © 2021 Salazar et al. 2021 Salazar et al. https://creativecommons.org/licenses/by/4.0/ This content is distributed under the terms of the Creative Commons Attribution 4.0 International license.

### Resistance is associated with loss of fitness in host microenvironments.

**FIG 2:**
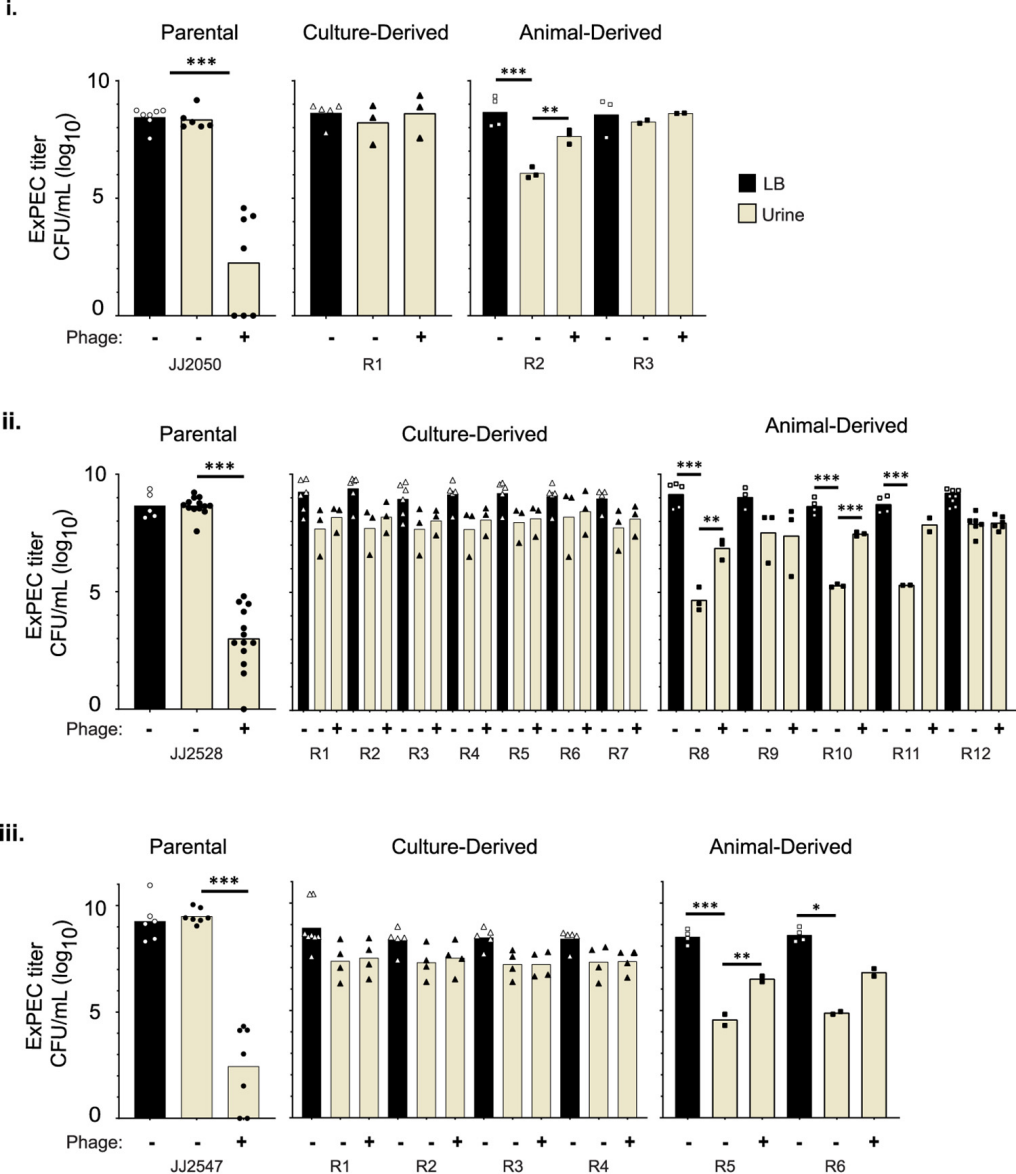
Resister survival in human urine. Isolate titers were determined in liquid culture after 4.5 h of incubation in LB (black) or human urine (yellow), with or without ϕHP3. Each panel is grouped by parental isolate: JJ2050 (i), JJ2528 (ii), and JJ2547 (iii). *P* values were determined by Student *t* test or Mann-Whitney test, where necessary. *, *P* < 0.05; **, *P* < 0.01; ***, *P* < 0.01. Each data point represents the average of three parallel technical replicates.

Resistance to phage may accompany a loss of fitness under certain environmental contexts; of particular interest are findings of loss of virulence in the hosts. Such a loss has been observed for bacterial pathogens of fish and moths, has been modeled in A. baumannii, and has been inferred with *V. cholera* in humans (24–29, 32–34). To assess virulence in our isolated resisters, we tested their growth ability in media that simulated the host microenvironment. Since ExPEC strains cause UTIs and bacteremia, human urine and blood were used. In urine, most of the resisters demonstrated comparable growth relative to LB; these same resisters likewise showed growth comparable to their WT progenitors (Fig. 2). Interestingly, all resisters with attenuated growth in urine (six of nine) were isolated from the murine model. Further, ϕHP3 retained effectiveness against WT isolates but was ineffective against resisters. Strikingly, and for reasons currently unknown, the six attenuated animal-derived resisters showed enhanced growth in the presence of purified ϕHP3 (Fig. 2; yellow bars, +). This growth enhancement was not replicated by the phage buffer alone (data not shown). Overall, these data suggest that most of the animal-derived resisters, though none of the culture-derived ones, have undergone a fitness trade-off that compromises their ability to grow well in human urine.

**FIG 3:**
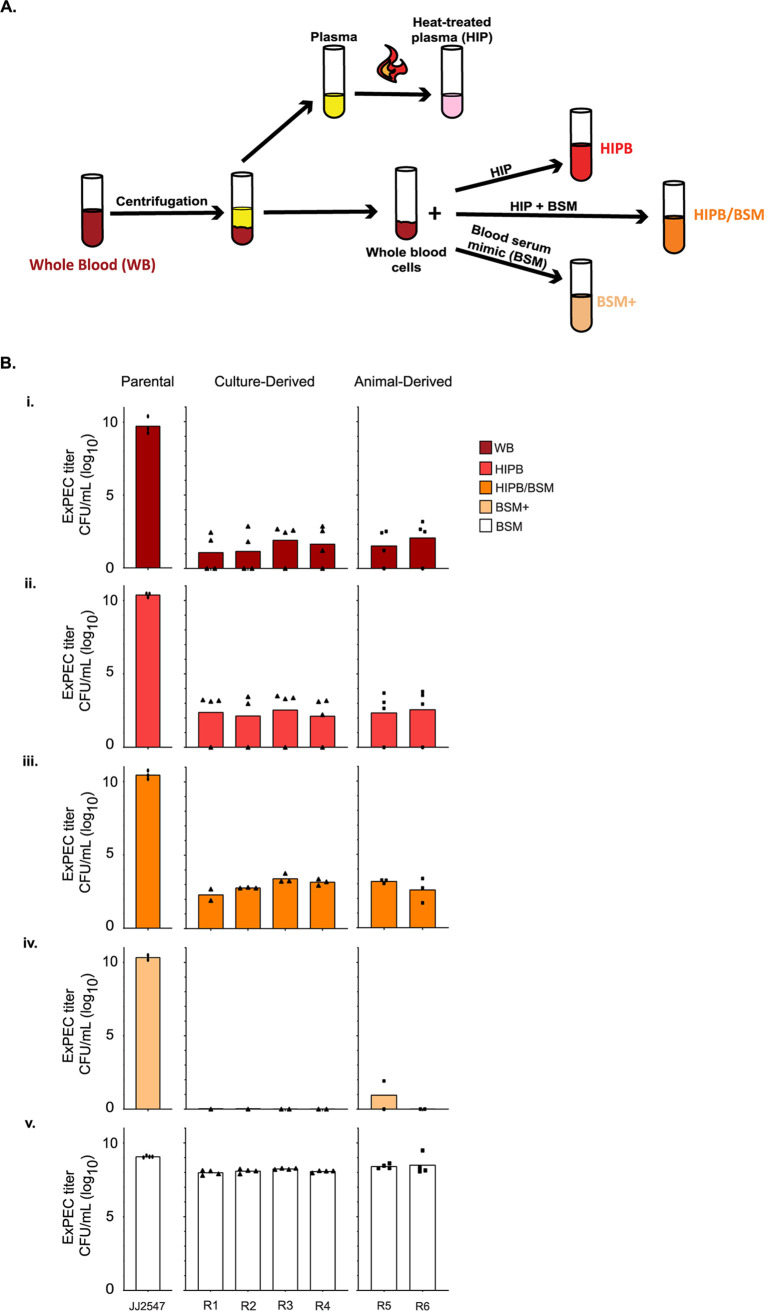
Resister survival in human blood. (A) Human whole blood was separated by centrifugation and the plasma heat-treated to inactivate complement. The whole blood cells (WBCs) were then resuspended in heat-treated plasma (HIP), blood serum mimic (BSM), or a 1:1 mix of HIP and BSM. (B) JJ2547 and resisters’ titers were determined after 24 h of incubation in different blood media: whole blood (WB, dark red) (i), HIP blood (HIPB, red) (ii), HIP/BSM blood (HIPB/BSM, orange) (iii), BSM (BSM+, light orange) (iv), or BSM with no blood cells (BSM, white) (v). *P* values were determined by Student *t* test or Mann-Whitney test, where necessary. *, *P* < 0.05; **, *P* < 0.01; ***, *P* < 0.01. Each data point represents the average of three parallel technical replicates.

To assess whether the ExPEC resistors demonstrated any fitness losses in blood, isolates were first assessed for growth and survival in unaltered human whole blood (WB; Fig. 3A). Whereas WT JJ2547 thrived in WB after 24 h, the resistor strains, regardless of isolation method, experienced significant decreases in viability (Fig. 3Bi). We hypothesized that the resisters’ fitness loss was due to the complement system in serum, a potent antibacterial mechanism. To disable complement, we heat treated the plasma fraction and recombined it with the blood cell fraction, a mixture termed heat-inactivated plasma blood (HIPB; Fig. 3A) (35). The resistor strains remained substantially attenuated, with an average 7-log drop in viability (Fig. 3Bii). We next hypothesized a loss in nutrient uptake may cause the resisters’ loss of fitness. To replicate the nutritional environment of blood in a more readily accessible form, we used a defined medium called blood serum mimic (BSM) (36). Resister survival was not improved when HIPB was mixed 1:1 with BSM (HIPB/BSM; Fig. 3Biii); interestingly, however, resister survival was poor when the blood cell fraction was suspended in BSM (BSM+; Fig. 3Biv). When grown in BSM alone, resister growth was comparable to the parental strain (Fig. 3Bv). This suggests that something in both the blood cell and the plasma fractions is unfavorable for resister survival. The other WT and resister strains reproduced this general trend (see Fig. S4). Interestingly, parental JJ2528 demonstrated a marked deficit in growth in blood; this was somewhat surprising due to the strain’s high virulence in a murine model of bacteremia (8, 30). Overall, these data suggest that in order for the resisters to overcome phage infection, they have acquired a dramatic loss of fitness in human blood that is not driven by complement or nutrient uptake.

10.1128/mBio.00211-21.4 FIG S4 Resister survival in human blood. Isolate titers were determined after 24 h of incubation in a series of mediums. Whole blood (WB, dark red), HIP blood (HIPB, red), HIP/BSM blood (HIPB/BSM, orange), or BSM (BSM+, light orange). *, *P* < 0.05; **, *P* < 0.01; ***, *P* < 0.01. (i) JJ2050 and resisters. (ii) JJ2528 and resisters. Each data point represents the average of three parallel technical replicates. The *P* values were determined by a Student t test or Mann-Whitney test, where necessary. *, *P* < 0.05; **, *P* < 0.01; ***, *P* < 0.01. Each data point represents the average of three parallel technical replicates. Download FIG S4, TIF file, 0.7 MB. Copyright © 2021 Salazar et al. 2021 Salazar et al. https://creativecommons.org/licenses/by/4.0/ This content is distributed under the terms of the Creative Commons Attribution 4.0 International license.

### Phage resistors are attenuated during systemic infection.

**FIG 4:**
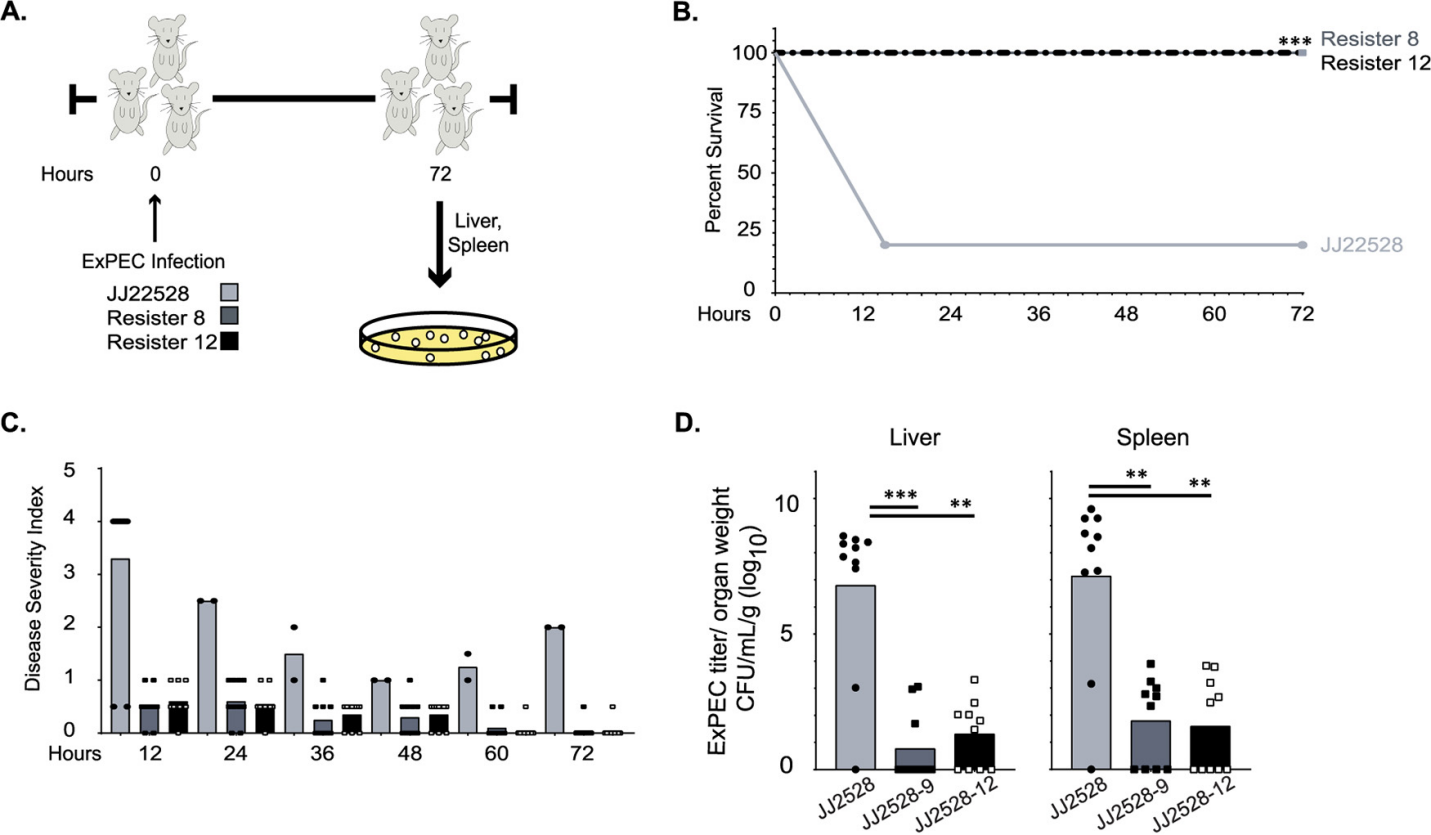
Virulence of resisters in murine sepsis. Two representative resisters, JJ2528-8 (dark gray, *ompA* truncated) and JJ2528-12 (black, *waaC* truncated), and their parental isolate (light gray) were tested for virulence in our murine sepsis model. (A) Swiss-Webster mice were infected with 3.5 × 10^7^ CFU log-phage cultures, suspended in PBS. Animals were observed for 72 h and then euthanized unless moribund. Livers and spleens were homogenized and plated to determine bacterial burden. (B) Animals infected with the parental isolate showed a survival rate of only 20%, whereas the resister-infected animals had a survival rate of 100%. (C) Infected animals were observed and given a health score using the four parameters outlined in the NIH Animal Research Advisory Committee Guidelines. A score of 4 or greater is considered moribund and requires euthanasia. (D) Livers and spleens of infected animals were collected after euthanasia, weighed, homogenized, and plated on LB to determine bacterial burden. Shown is the titer per gram weight. The *P* values in panel B were determined by log-rank (Mantel-Cox) test. *P* values in panel D were determined by a Student *t* test or Mann-Whitney test, where necessary. *, *P* < 0.05; **, *P* < 0.01; ***, *P* < 0.01.

The resisters’ loss of fitness in human blood and, in several of the animal-derived resisters, urine prompted us to assess resister pathogenicity in a murine model of bacteremia (Fig. 4A). For this study, we selected two representative isolates from our resister list (both animal-derived, based on colony morphology) and compared their virulence and bacterial levels to the parental strain after infection. Surprisingly, every animal infected with the resister strains survived for the duration of the experiment compared to 20% survival with the parental strain (Fig. 4B). Further, animals infected with the resisters had lower disease severity scores at every time point of the study (Fig. 4C) and displayed, on average, an ∼4.5-log reduction in organ bacterial burden (Fig. 4D). These data suggest that these resisters sustained a loss of fitness in a murine model of systemic infection, consistent with the observed loss of survival in simulated host microenvironments.

### Mechanism of resistance relates to mutations in bacterial surface components.

**FIG 5:**
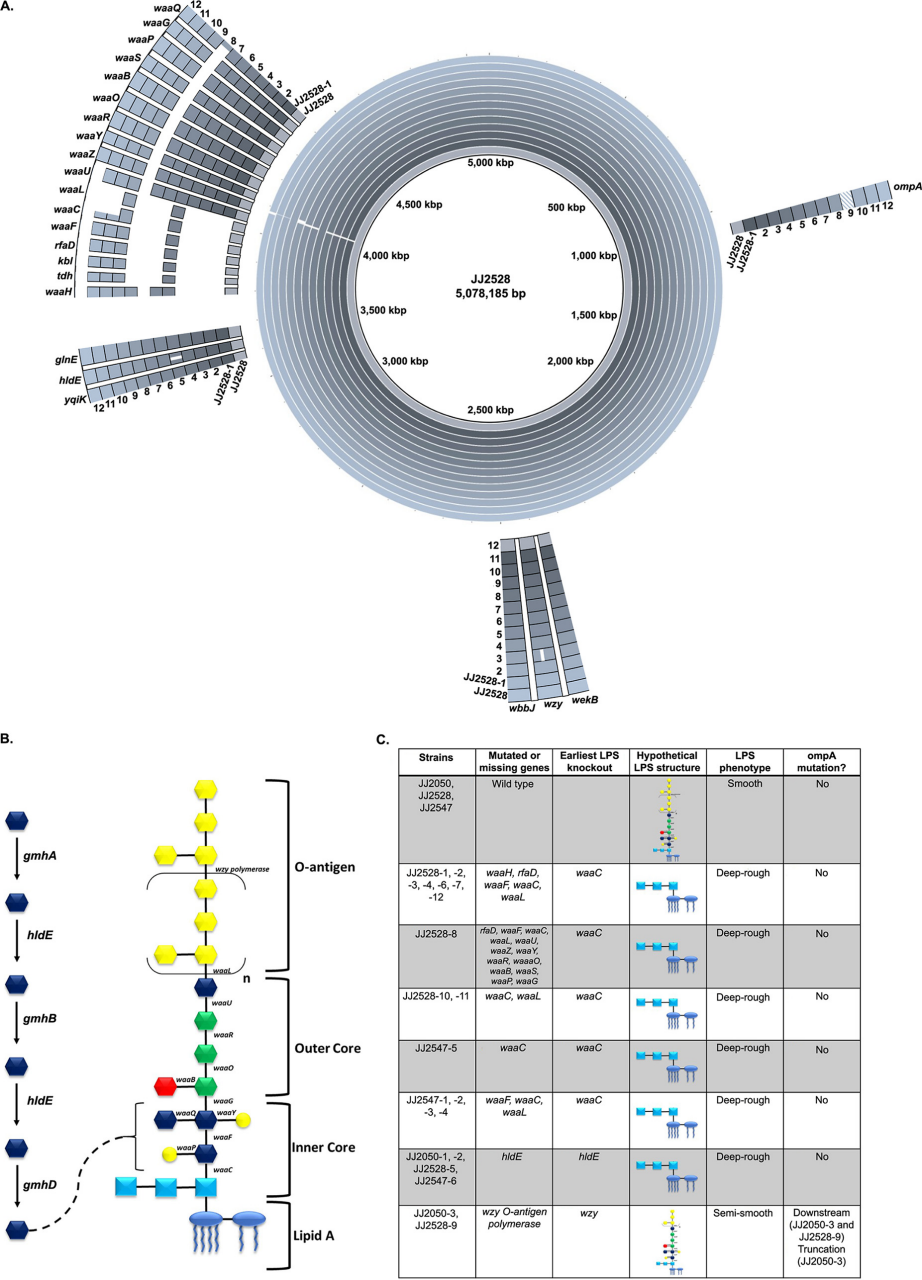
Whole-genome sequencing of isolates. The genomes of all 21 resisters and their parental isolates were sequenced. (A) JJ2528 and its resistant isolates were aligned to reveal several truncations of interest, particularly in the *waa* operon, *hldE*, and *ompA*. Dashed fill for panel indicates the downstream SNP in *ompA*. (B) Structure of LPS, naming the enzyme responsible for forming each linkage. Also shown are several steps in the construction of LD-Hep (left).

To understand the mechanism driving ExPEC resistance to ϕHP3, as well as give insight into the reasons for reduced virulence, whole-genome sequencing was performed on all 21 resisters, followed by an alignment of the assembled genomes to the parental strains (Fig. 5A; see also Fig. S5). Remarkably, 15 of 21 resisters, regardless of the parental strain or isolation strategy, harbored truncations of various degrees in a single operon: the *waa* (or *rfa*) system, which is responsible for the assembly of lipopolysaccharide (LPS) (37). Of these 15, one gene, *waaC*, was truncated or missing in every mutant. WaaC is responsible for the attachment of the second saccharide (l-glycero-d-manno-heptulose [LD-Hep]) to the first (1-deoxy-d-manno-oct-2-ulsonic acid [KDO]), forming the inner core of LPS (Fig. 5B). This mutation likely results in loss of most of the inner and outer core, as well as O antigen. Four of the remaining six resisters had truncations in *hldE*, responsible for two steps in the synthesis of LD-Hep (Fig. 4B). This mutation, phenotypically, is likely to produce a truncated LPS molecule identical to the *waaC* truncation. This is consistent with the findings of Mutalik et al. (38), who found a tendency for E. coli strains to develop mutations in the *waa* operon or in the construction of LD-Hep when developing resistance to coliphages. This was similarly found for isolates of Pseudomonas aeruginosa (39). This observation points to a conserved mechanism of resistance for ST131 E. coli to ϕHP3 via the truncation of surface LPS.

10.1128/mBio.00211-21.5 FIG S5 Whole-genome sequencing of isolates. The genomes of all 21 resisters and their parental isolates were sequenced. (A) Alignment of JJ2050 and its resistant isolates. (B) Alignment of JJ2547 and its resistant isolates. Download FIG S5, TIF file, 1.7 MB. Copyright © 2021 Salazar et al. 2021 Salazar et al. https://creativecommons.org/licenses/by/4.0/ This content is distributed under the terms of the Creative Commons Attribution 4.0 International license.

Of the final two resisters, one had a truncation in *ompA*, which expresses outer membrane protein (OMP) A, which forms pores in the bacterial outer membrane to import nutrients. Both of the two remaining mutant strains had a single nucleotide polymorphism (SNP) shortly downstream from the gene of *ompA*, which we hypothesize to be a rho-independent terminator region (40); this modification may affect transcript polyadenylation, thus decreasing transcript stability (41–44). These two resisters also had truncations in the *wzy* O-antigen polymerase gene, although this may be unrelated to phage resistance in this case. A summary of the mutations in each of the 21 resisters, as well as a structural representation of their location in the LPS molecule, is shown in Fig. 5C. These findings suggest that the ST131 resister isolates attained resistance to ϕHP3 through loss of either LPS (19/21 cases) or OmpA (2/21 cases). Since both of these components are located on the surface of E. coli, the data suggest that these two features may constitute primary and/or secondary receptors for phage HP3. An alternative hypothesis is that the loss of these genes disrupts E. coli’s surface integrity in a way that prevents proper attachment or adsorption of HP3. These possibilities are addressed in the Discussion.

### Directed evolution guides the emergence of antiresister phages.

A somewhat unexpected finding from this work was the frequency at which ST131 resisters arose *in vitro* and during infection and their convergence in all 21 independent cases toward one or two key mutations in LPS or OmpA. Although there was clear reduction in virulence in the two mutants tested by murine sepsis model (one mutant in LPS and one in OmpA; Fig. 4), it is worth noting that they were isolated during phage challenge of parental strains in infected animals and were present at high enough levels to be isolated (Fig. 1). The consistency of these findings suggests that a highly effective, “two-hit” phage cocktail could be developed, one specifically formulated with phages to drive development of resisters that are less virulent in their host, and phages (developed to “predict” resistance mechanisms) which target those resisters.

**FIG 6:**
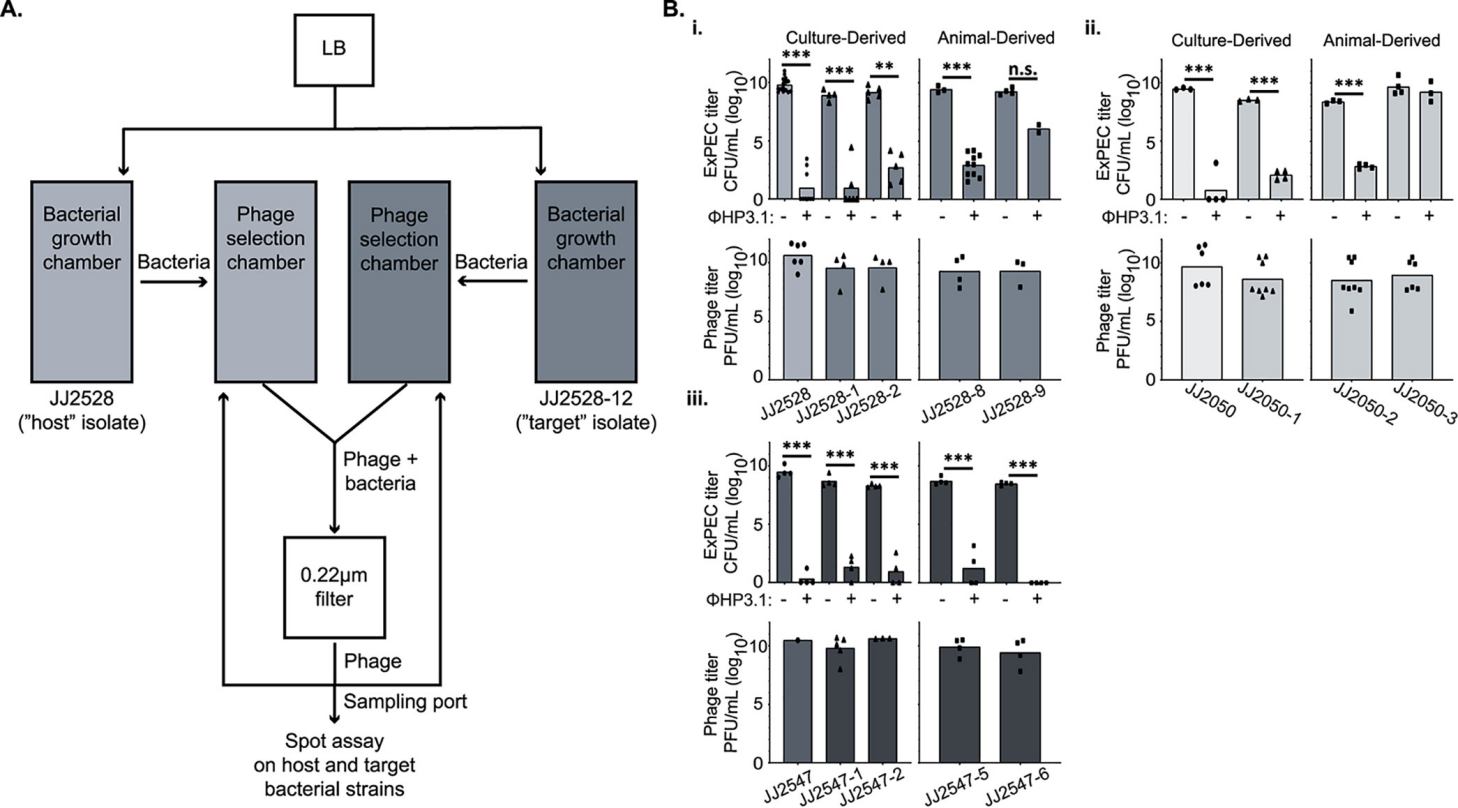
Evolution of ϕHP3. (A) Resister JJ2528-12 was used as a target for the evolution of ϕHP3. ϕHP3 was allowed to adapt to the target in a continuous-flow bioreactor, in a method similar to the Appelman’s protocol. (B) Isolate titers were determined in liquid culture after 4.5 h of incubation in LB with or without the presence of the evolved ϕHP3.1 (top panels). The ϕHP3.1 titer was also determined on these isolates using a standard phage spot assay (bottom panels). Panel B is grouped by parental isolate: JJ2050 (i), JJ2528 (ii), and JJ2547 (iii). The *P* values were determined by a Student *t* test or Mann-Whitney test, where necessary. *, *P* < 0.05; **, *P* < 0.01; ***, *P* < 0.01. Each data point represents the average of three parallel technical replicates.

To test this, we first screened two well-characterized phages (ϕES17 and ϕEC1) for their ability kill the ST131 resister strains (45, 46). Surprisingly, although these phages effectively kill all three parental strains, every resister was refractory to killing by these phages (Table 2). That these phages are distinct from HP3 (genetically and morphologically) may suggest that there are common mechanisms of resistance in ST131 to coliphages. A screen of sewage samples active on parental strains on resister lawns also did not reveal any candidate lytic phages against the resisters (data not shown). We hypothesized that the original phage, ϕHP3, could undergo adaptation in order to reinfect the resisters. To test this, we generated an automated bacterium-phage bioreactor that continuously cycles fresh phage grown on its original bacterial host (parental strains) into a chamber that contains the target bacterium (resistant isolates) (Fig. 6A). We predicted that automated continuous coculture cycles of infection and phage production on the parental host will lead to a rare phage variant (mutant) that can “jump” or adapt to the target resister strain. This principle is similar to the original Appelman’s protocol for facilitating growth of phage on hosts refractory to predation, except that the version developed here is automated and continuous (47). A description of the methods of this technology is provided in the supplemental material.

**TABLE 2:**
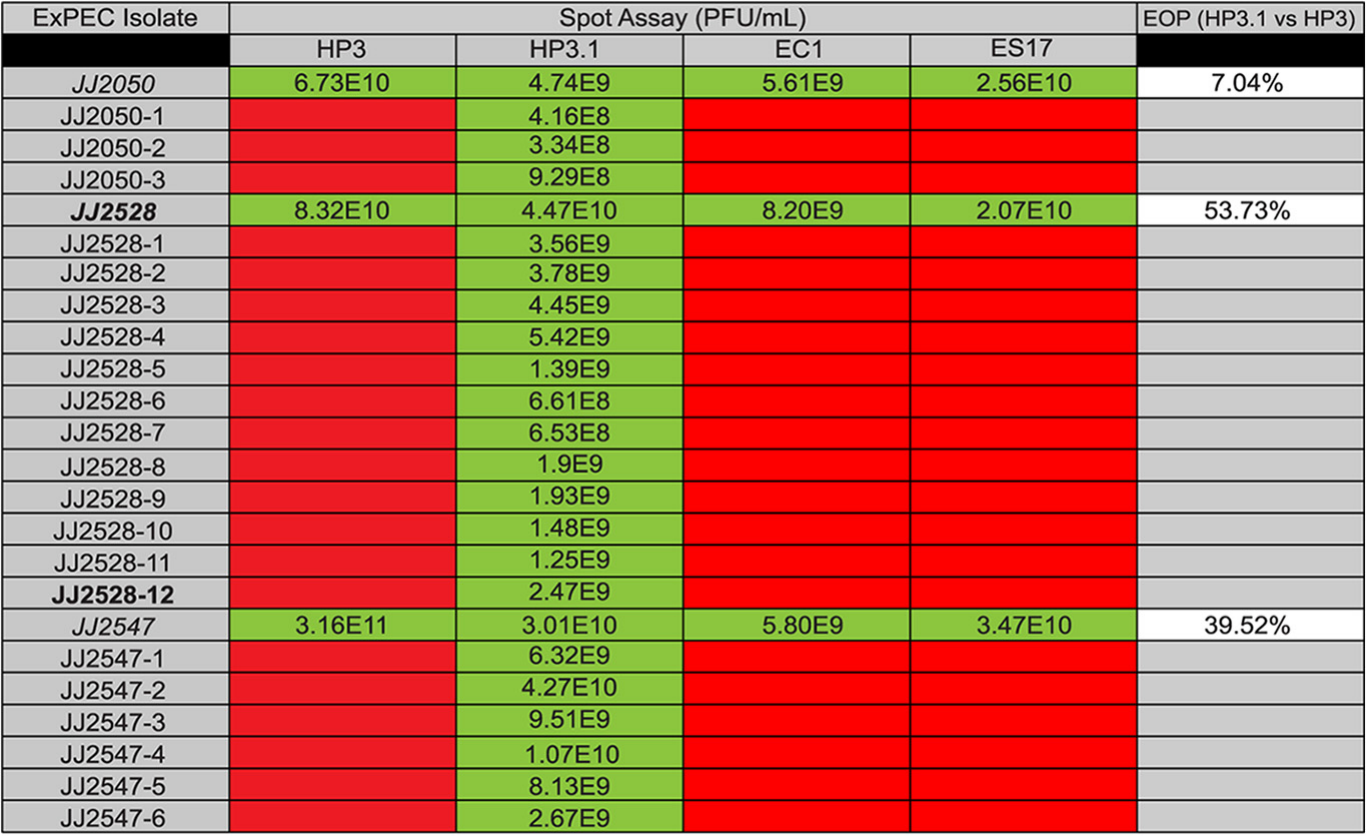
Phage screen against resistant isolates^*a*^

a Resisters were screened against ϕHP3, ϕHP3.1, ϕEC1 (similar to ϕHP3), and ϕES17 (dissimilar to ϕHP3) by phage spot assay. The phage titer is shown, where applicable. Green squares indicate plaque formation, and red squares indicate resistance to the phage. The ϕHP3.1 efficiency of plating is shown relative to that of ϕHP3 on parental ExPEC isolates

To determine whether this approach would yield a phage derivative capable of infecting the resisters, parental JJ2528 was cocultured with phage ϕHP3, and phage progeny then cycled to the chamber containing the resister isolate JJ2528-12. Samples were taken from the chamber at 5, 21, 24, and 37 h and tested by spot assay on both the host and target isolates (see Fig. S6). Unexpectedly, a sample taken at 5 h yielded noticeable clearing on JJ2528-12, as well as the parental host, which increased with time. A plaque from these was isolated, expanded in its new host, and purified. The new phage, designated ϕHP3.1, showed bacterial killing in the liquid culture assay (and plaque formation in the spot assay) for most of the resisters (either animal or culture derived) of JJ2528 (Fig. 6Bi). In addition, ϕHP3.1 was able to infect not just the target resister used in the directed evolution experiment but also every LPS-truncated resister, while retaining effectiveness against all three parental isolates (Fig. 6Bii and iii; Table 2). Interestingly, the two *ompA* resisters retained partial (SNP only, JJ2528-9) or full (SNP and truncation, JJ2050-3) immunity to the evolved phage.

10.1128/mBio.00211-21.6 FIG S6 Evolution of ϕHP3. Samples of the circulating phage were collected at hours 0, 5, 21, 24, and 37. All time points were tested by spot assay on the parental (JJ2528) and target (JJ2528-12) strains. Faint clearing can be seen starting at h 5. Download FIG S6, TIF file, 0.5 MB. Copyright © 2021 Salazar et al. 2021 Salazar et al. https://creativecommons.org/licenses/by/4.0/ This content is distributed under the terms of the Creative Commons Attribution 4.0 International license.

**FIG 7:**
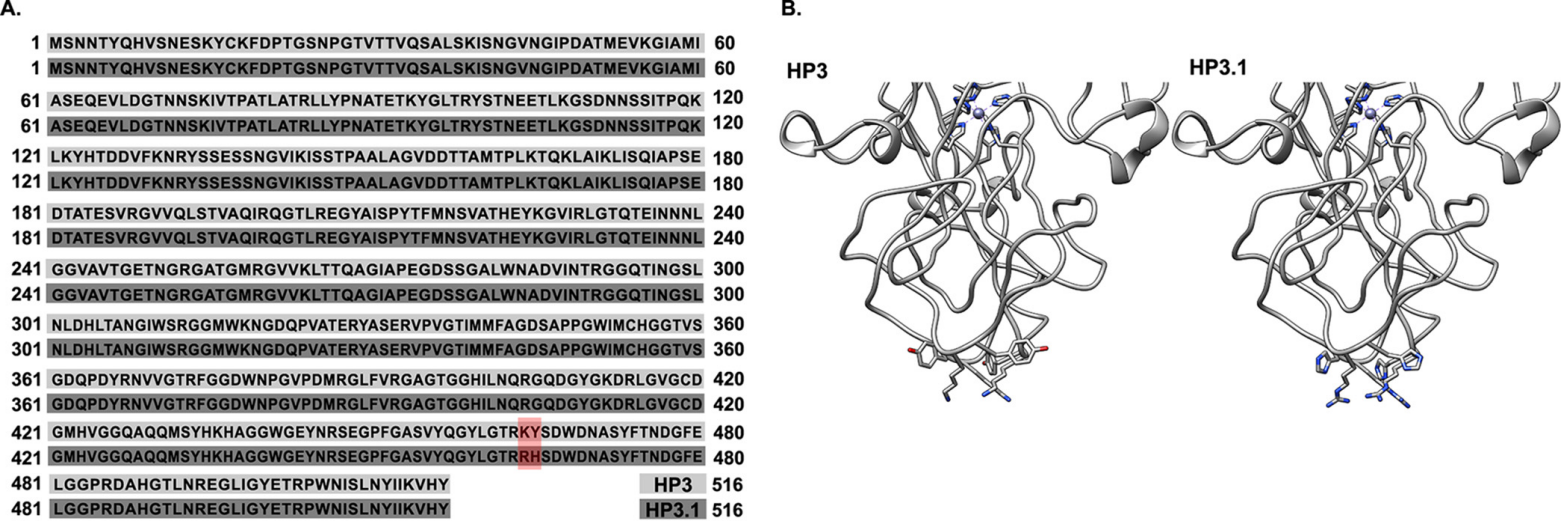
Sequencing of ϕHP3.1. (A) Sequencing revealed a key missense mutation in the binding spike of ϕHP3.1 (red highlight). (B) Residue substitutions were determined to be located at the binding region of the tail protein by protein modeling, based on the structure of the tail spike of ϕT4.

To verify ϕHP3.1 was a derivative of ϕHP3, and to identify genetic changes associated with its broad activity against the resisters, we subjected the purified phage to whole-genome sequencing. Bioinformatic comparison of ϕHP3.1 to ϕHP3 indicated just two SNPs along the length of all 176,000 bp. The first of these changes, a LysTyr464 to ArgHis464, was located in the gene encoding the spike protein (Fig. 7A), potentially at its binding site with the bacterial host (Fig. 7B). The second SNP coded a missense mutation in the long tail fiber gene, Gln9 to Arg9. Both instances result in substitution of residues with nonpolar side groups to ones with positive charges at the extreme tip of the spike gene. This suggests that phage ϕHP3.1 may be able to reinfect ST131 resisters through compensatory mutations which enhance the phage’s interaction with the host surface, possibly via electrostatic charge modifications that promote binding or adsorption.

### Compensatory ST131 ExPEC mutations allow resister reresistance to the evolved ϕHP3.1.

**FIG 8:**
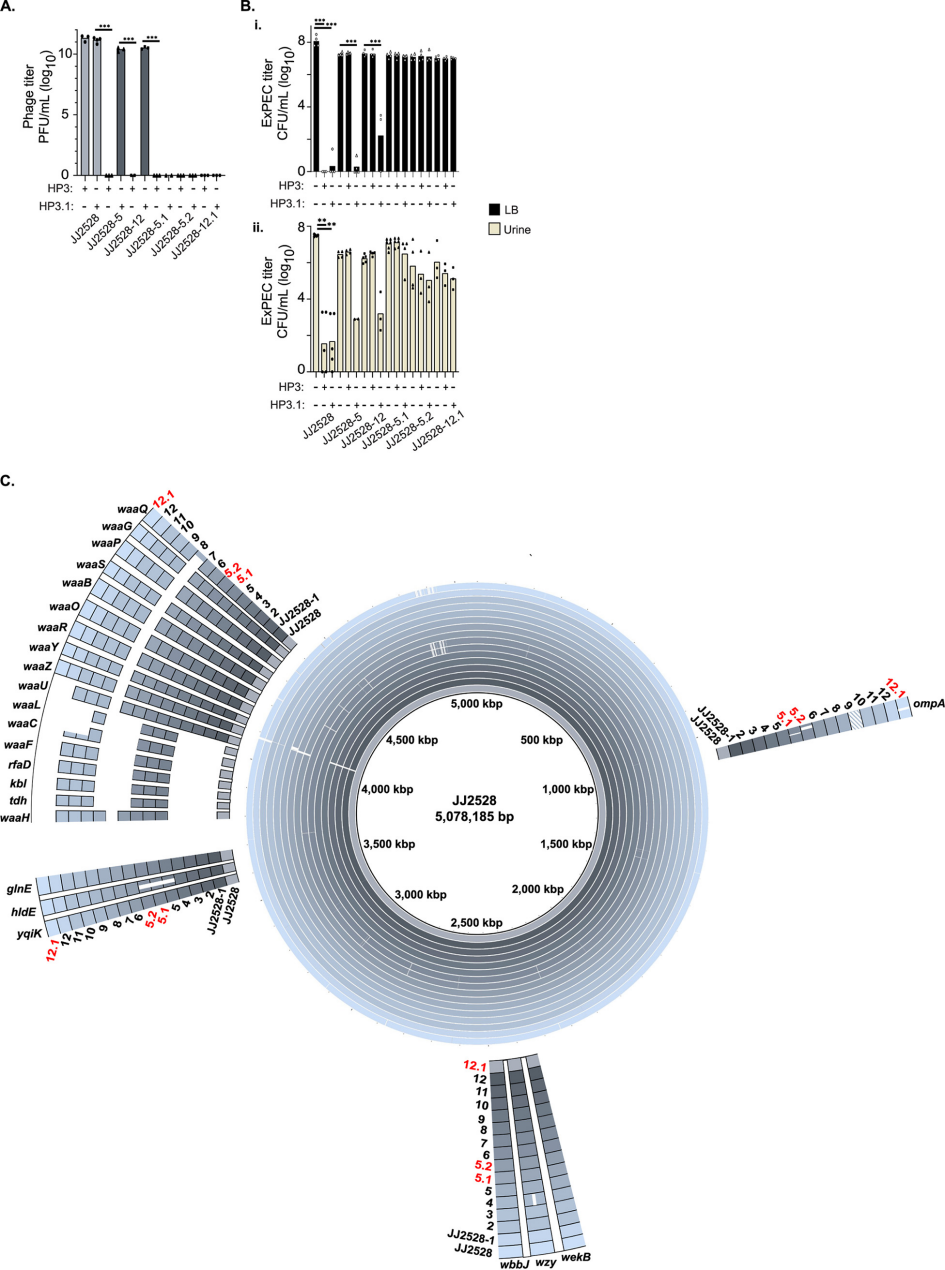
Secondary, ϕHP3.1-resistant isolates. Two ϕHP3 resisters, JJ2528-5 and JJ2528-12, were used to select ϕHP3.1-resistant isolates using the culture-derived method depicted in Fig. 1B. (A) Phage (ϕHP3 and ϕHP3.1) titers were determined by spot assay on the parental isolate and primary and secondary resisters. (B) Isolate titers were determined in liquid culture after 4.5 h of incubation in LB (black) or urine (yellow), with or without the presence of either ϕHP3 or ϕHP3.1. (C) Whole-genome sequencing was performed on the secondary resisters, and they were aligned with the parental isolate and primary resisters. The secondary resisters retained truncations in the *waa* operon and acquired truncations in *ompA*. A dashed fill for panel indicates the downstream SNP in *ompA*. The *P* values in panels A and B were determined by a Student *t* test or Mann-Whitney test, where necessary. *, *P* < 0.05; **, *P* < 0.01; ***, *P* < 0.01. Each data point represents the average of three parallel technical replicates.

Phage and their bacterial hosts engage in cycles of coevolution (18, 21, 22, 48). We wondered whether the resister strains had the ability to once again evolve resistance against the new phage, ϕHP3.1. Using the culture-derived selection method depicted in Fig. 1A, we identified three ExPEC isolates that grew in the presence of ϕHP3.1: two from JJ2528-5 (JJ2528-5.1 and JJ2528-5.2) and one from JJ2528-12 (JJ2528-12.1). It was not possible to isolate animal-derived resisters to HP3.1 due to their poor virulence in infection challenge models (Fig. 4). The new resister strains were refractory to killing in both phage spot assays (Fig. 8A) and liquid culture assays (Fig. 8Bi), confirming their resistance. These secondary resisters maintained their ability to grow in human urine (Fig. 8Bii). Since the parental resisters were already not viable in human blood (see Fig. S4ii), we did not test the new resisters in this medium. Whole-genome sequencing of these resisters showed they maintained their parental truncations in *hldE* and *waaC*, respectively. Rather excitingly, and consistent with the results presented above, all three acquired new truncations in their *ompA* genes, similar to JJ2050-3 (Fig. 8C). This finding strongly suggests that OmpA is a partial receptor for phage ϕHP3 and a primary receptor for its evolved progeny, ϕHP3.1. That the *ompA* modification was observed in two of the 21 original resisters may reflect phage-ST131 evolution dynamics occurring at this second step during the first screen, possibly due to added selective pressure from the murine system.

## DISCUSSION

This report addresses knowledge gaps regarding mechanisms of phage resistance during phage therapy. We observed here a number of important features regarding this resistance. (i) The pandemic E. coli strain ST131 can become resistant to a previously used therapeutic phage (ϕHP3), both *in vitro* and during phage therapy. (ii) Of 21 resisters, across three different strains and two different selection criteria, 19 harbor mutations in genes encoding the system which synthesizes LPS. The remaining isolates harbored a mutation in OmpA. (iii) The resisters were able to grow in human urine but were substantially compromised in human blood. (iv) The resisters were avirulent in a murine model of bacteremia. (v) A bioreactor system that facilitates the continuous coculture and cycling of ϕHP3 between susceptible and resister hosts yielded a new mutant phage (ϕHP3.1) that efficiently lyses all the LPS-truncated resisters, as well as their parents. (vi) ϕHP3.1 harbors two point mutations between the putative long and short tail fibers, presumably enhancing the binding of this phage to the resisters. (vii) Finally, resistance of the isolates to ϕHP3.1 is possible through the acquisition of a secondary mutation in the gene that encodes OmpA.

Bacterial hosts will develop resistance to their phage predators. During the development of resistance, these isolates may be forced to exchange fitness in other environments (9, 24, 26, 27). Although resisters have been developed in culture and found to have attenuated infectivity, phage resisters have yet to be isolated and characterized from a therapeutic setting (9, 28, 29, 32–34). Further, these studies are largely limited to pathogens with nonmammalian hosts. One exception is by Altamirano et al. (29), who found a loss of virulence in phage-resistant A. baumannii in a murine infection model. The other exception is by Seed et al. (27), who found loss of virulence in some Vibrio cholerae strains isolated from cholera patients. These patients were not treated with phage, however, and resistance was to the patients’ native phage population. To address this gap in knowledge in a controlled, therapeutic setting, we used a previously developed murine sepsis model in parallel with a more traditional approach to select for phage-resistant isolates from clinically derived pathogenic E. coli. Under both *in vitro* and *in vivo* conditions, the three parental ExPEC isolates (JJ2050, JJ2528, and JJ2547) developed resistance to ϕHP3. We isolated a total of 21 resisters, 12 from culture and 9 from mice. Both methods were performed in parallel with phage-untreated controls; that no resisters emerged from phage-free conditions strongly implies that the resisters developed as a direct result of selective pressure.

A major draw of phage therapy is the rapid adaptability of phages to new targets. Phages exist on a spectrum between two groups: the first have very narrow host ranges and tend to have high infectivity in their targets, and the second have wider host ranges and lower infectivity in any specific target. It has been found that phages can be “trained” to move in either direction depending on the hosts to which they are exposed (18, 21, 22, 25, 39, 49, 50). Our results support this notion. Using a modified bioreactor, we evolved ϕHP3 against the resister JJ2528-12 and isolated a mutant phage. We found that ϕHP3.1 retained virulence against all three parental ExPEC isolates, but at a lower efficiency of plating. Excitingly, however, we found that it was also able to infect the other 18 LPS-truncated resisters. The sensitivity of resisters to the new phage is significant because we found that they were resistant not only to the parental phage but also to other, unrelated phages. This finding suggests that generalized forms of resistance may be widely protective against phages; however, new phages isolated by directed evolution may overcome these barriers and be capable of infecting a wide range of resistant hosts.

Mechanisms by which bacteria achieve phage resistance are still being discovered, but it is generally accepted that some are more pervasive than others. In particular, common methods by which bacteria prevent infection after phage binding include restriction-modification (R-M) and CRISPR-Cas systems, which identify and destroy foreign DNA (23, 51–53). Some bacteria containing prophage genes prevent infection through superinfection exclusion (Sie) systems (23, 52, 53). Other bacteria prevent phage adsorption all together, commonly by blocking, modifying, or even eliminating the phage’s receptor (23, 52, 53). The latter mechanism is the most likely for the resisters isolated in this study. Sequencing of the three parental isolates used here revealed they are lacking functional Cas and have little in the way of R-M systems. Further, we found highly consistent truncations across the primary (ϕHP3) and secondary (ϕHP3.1) resisters in the *waaC* and *ompA* genes. The presence of these truncations, rather than SNPs, is notable because they have a low chance of reversion; it is possible the truncations were necessary for stable phage resistance. The resistance of the primary and secondary resisters with *ompA* mutations against both phages strongly points to the OmpA protein as a receptor, in some capacity, of both phages. This is supported by earlier studies, which have found that OMPs, including OmpA, serve as both receptors for coliphages and as phages of other species (38, 54–58). It is known that LPS plays a vital role in the folding and placement of outer membrane proteins of the cell (59–64). In particular, it is known that LPS works with Skp to place OmpA in the outer membrane and helps it develop its secondary and tertiary structure (24, 38, 52, 61, 65). One possibility is that the loss of LPS prevents OmpA from being properly positioned in the outer membrane, a condition which may result in resistance to phage. That mutations in OmpA were found on all reresisters derived against HP3.1 supports this notion. Under this model, it would seem that it is easier for LPS to be mutated then OmpA (or that the loss of LPS is less harmful then OmpA). This may explain why, in the first round, most resisters first acquired mutations in LPS synthesis. However, we cannot yet exclude that LPS and OmpA do not form as some type of a coreceptor or secondary receptor complex for the phage. More work will be needed to make this determination.

We observed that when ST131 becomes resistant to phage, it attenuates its virulence in a murine infection model. This may be through a substantially reduced ability to survive in blood. Gram-negative bacteria with LPS truncated at the inner core are referred to as deep-rough mutants (66, 67). Studies have found that they are significantly more permeable to lipophilic and anionic compounds than are their smooth and rough counterparts. This is because they lack crucial stabilization provided by two forces: a cationic “cloud” (Ca^2+^ and Mg^2+^) drawn in by the negatively charged LPS and, to a lesser extent, hydrogen bonds. Intact, this forms a somewhat rigid, charged barrier between the cell and its environment, allowing only some small, cationic molecules through (37, 63, 67–71). Consistent with these findings, our *waaC-* and *hldE-*truncated isolates were unable to survive when challenged with sodium dodecyl sulfate (SDS), and anionic compound which has no effect on the WT or *ompA-*truncated isolates (see Fig. S7). It is known that immune factors in blood are subject to interference by the outer membrane of Gram-negative pathogens; for example, defensins are inhibited by cations. It is tempting, then, to speculate that the loss of these factors permeabilizes the outer membrane to charged (here negative) or lipophilic small molecules, causing the attenuation we see in blood and infection.

10.1128/mBio.00211-21.7 FIG S7 Resister survival with SDS. Isolate titers were determined in liquid culture after 4.5 h of incubation in LB in the presence of SDS at 10, 100, and 200 μg/ml. Shown is the percent survival relative to growth in the absence of SDS. Resisters were selected so that each parental isolate and each truncation (*waaC*, *hldE*, and *ompA*) was represented twice (noted on *y* axis label). *, *P* < 0.05; **, *P* < 0.01; ***, *P* < 0.01. Each data point represents the average of three parallel technical replicates. Download FIG S7, TIF file, 0.9 MB. Copyright © 2021 Salazar et al. 2021 Salazar et al. https://creativecommons.org/licenses/by/4.0/ This content is distributed under the terms of the Creative Commons Attribution 4.0 International license.

We developed a continuous cycle coculture bioreactor to test the notion that ϕHP3 can be adapted to the ST131 resister strains. The premise is similar to the Appelman’s protocol. A phage or cocktail is allowed to replicate in a series of susceptible (host) and resistant (target) bacterial isolates; wells with bacterial clearing are pooled, and the process is repeated with the pooled phage until a satisfactory number of the isolates are cleared (47). Our bioreactor builds on this concept and automates the process. The bacterial host and target are kept in separate chambers which have a continuous flow of fresh media. The selected phage is continually cycled between the two, thus replicating the act of allowing phage expansion, pooling, and reexpansion. With this process, we observed in just 5 h the emergence of a phage that formed plaques on the resisters and termed this phage HP3.1. Whole-genome sequencing revealed only two mutations facilitated this conversion, both of which conferred a net-positive charge, as determined by modeling, of the putative phage spike proteins. This is of particular interest since the truncation of LPS at the inner core eliminates all surface phosphorylated saccharides, significantly decreasing the net negative charge. This suggests that the increase of positive charge at these regions play a role in phage-host interactions through the compensation of electrostatic interaction.

In addition to the finding that a continuous bioreactor system can facilitate the selection of a new phage that targets ExPEC resisters, two additional findings of high importance are reported here. The first, as discussed above, is that all resisters acquired mutations in just two pathways (LPS biosynthesis and OmpA expression). This suggests that, despite a plethora of possible (and diverse) mutations that can confer resistance to a coliphage, ST131 is locked into only two key evolutionary trajectories that can produce functional escape of a phage challenge. Although these findings suggest that we can predict phage-pathogen interactions, this study is limited in scope and more research, branching into other phages and bacteria, is necessary. This bodes well for phage therapy and suggests that it may be possible to make a cocktail of only a few phages: one that targets the original host and one or two that target the resisters that may result. Two studies found that by developing a cocktail of two phages, one parental and one resister adapted, they could drastically decrease the development of resisters in comparison to the parental phage alone (39, 72). It has also been found, however, that bacteria challenged with a cocktail may be more likely to develop “generalized” mechanisms of resistance, giving them a broader resistance to phage and making them more difficult to treat with other phages (73). It is thus worth exploring whether there is an ideal timing in the application of phages, thereby forcing the population to “commit” to specific mechanisms of resistance before applying resister-adapted phages.

Second, and unexpected here, just as predicting the mechanism of resistance is vital to phage therapeutics, the fitness consequences of phage-resistance can give us further insight on how to exploit these pathogens. In a laboratory setting, bacteria are generally kept under ideal conditions; this conceals potential fitness losses that may prove fatal in the context of host infection. To explore the fitness of the isolated resisters, we performed *ex vivo* growth assays in human urine and blood. Several of the animal-derived resisters showed attenuated growth in urine. There is no obvious link between this phenotype and resistance mutation (LPS/*ompA* truncation), suggesting a different mutation is responsible. This loss of fitness is likely the result of increased selection pressure in an infection setting and not present in culture conditions. In blood, we found a universal failure of the resisters to thrive. Every resister, regardless of parental strain or isolation strategy, showed a significant or complete loss of countable bacteria in each blood mixture after 24 h of incubation. In the absence of whole blood cells (WBCs), resisters similarly died in heat-treated plasma and complement (C3)-depleted serum (data not shown). In synthetic BSM, however, they showed similar titers to LB. This complete loss of viability in blood is particularly striking in the animal-derived resisters, considering that they were isolated from a model bacteremia. Because the resisters die in all mixtures containing WBCs, it is reasonable to hypothesize that a factor in the blood is killing the resisters. This may be facilitated by the increased permeability of most of the resisters due to truncated LPS (see Fig. S7).

It has been shown by Roach et al. (74) that there is cooperation between a host’s immune system and the phage which changes the course of a bacterial infection. To determine whether this loss of fitness corresponded to a loss of virulence, we selected two resisters, JJ2528-8 (*ompA* truncated) and JJ2528-12 (*waaC* truncated) to use in our murine sepsis model. We observed 100% survival of mice infected with a titer that was 80% lethal with the parental isolate. In addition to survival, the resister-infected mice were healthier overall and had significantly lower bacterial counts in their organs at 72 h postinfection. Although murine immune systems are vastly different from those of humans, combining this finding with the loss of fitness in human blood strongly points to a loss of virulence in these resisters with regard to a human host. Thus, it seems that in addition to a limited number of possible mutations which support escape from phage, the few mutations that do occur render the resulting ST131 strains attenuated in mammalian hosts. These findings raise the possibility that antibacterials of the future may have “predictive” attributes that not only leverage their ability to kill strains that evolve along a defined mutational pathway but also guide the microbes’ evolution into an outcome that either the human host or another phage can readily handle.

## MATERIALS AND METHODS

### Bacterial strains and growth conditions.

ExPEC strains JJ2050, JJ2528, and JJ2547 were used as parental isolates to develop phage resisters. These strains were kindly provided by James R. Johnson (6). In every case, these strains were grown overnight from a single colony in Luria broth (LB; Sigma-Aldrich) at 37°C and 250 rpm.

### Bacteriophage preparation.

ϕHP3 was isolated from waste samples collected at a local park (8). Phages were expanded in a host (JJ2528) bacterial strain in increasing volumes of culture in LB. The final batch was precipitated with CsCl (Sigma-Aldrich), and the band was collected after ultracentrifugation for 18 h. Band identities were confirmed by determining the index of refraction and dialyzed in phage buffer. The phage buffer was composed of 100 mM NaCl, 6.7 mM Tris-HCl, 3.2 mM Tris base, 10 mM MgSO_4_·7H_2_O, and sterile water. The buffer was brought to pH 8. All components were purchased from Sigma-Aldrich.

### Experimental animals.

Animal methods described were approved in accordance with appropriate guidelines and regulations by Baylor College of Medicine’s Institutional Animal Care and Use Committee. All mice were Swiss-Webster (Charles River, Wilmington, MA), female, and 6 weeks old at the time of infection. The animals were kept in filtered cages and had free access to sterile food and water.

### Culture-based resister selection.

Ciprofloxacin (Sigma-Aldrich) was suspended in LB top agar at a concentration of 10 μg/ml. We selected this antibiotic because, in addition to all three parental strains being resistant, it should not affect the outer membrane, minimizing its risk of interfering with phage-bacterium surface interactions. Once solid, 10^8^ PFU of ϕHP3 (8, 30, 31) was spread on the plate and allowed to dry. Finally, 10^7^ CFU of overnight culture were spread on the same plate, followed by incubation overnight at 37°C. The multiplicity of infection (MOI) was 10.

### Animal-based resister selection.

Mice were infected according to the method described by Green et al. (8), using three mice per group. Bacterial cultures were grown overnight. Subcultures were grown to log phase the next day and washed twice before suspension in phosphate-buffered saline (PBS; Sigma-Aldrich). A dose of 10^8^ CFU, confirmed by plating, was administered by i.p. injection. An hour after infection, phage-group mice were administered 10^9^ PFU of ϕHP3 (in phage buffer) at an MOI of 10 by i.p. injection. Health scores were assessed using the four parameters outlined in the NIH Animal Research Advisory Committee Guidelines (75). Mice were euthanized 15 h postinfection, and the livers and spleens were collected. The organs were hand homogenized with 0.5 ml of PBS, and 100 μl was spread on LB plates, which were incubated overnight at 37°C.

Resister virulence assays were performed in the same way with the following deviations: (i) ten animals were used per group; (ii) animals were infected with 3.5 × 10^7^ CFU of log-phase bacterial culture (none were treated with phage); and (iii) animals with acceptable health scores were taken to an endpoint of 72 h postinfection, at which point the survivors were euthanized.

### Liquid culture assay.

Overnight bacterial cultures were diluted to an optical density at 600 nm of 0.001 before being added to the selected media, with or without phage, to a final volume of 150 μl. The final bacterial concentration was 6.5 × 10^4^ CFU/ml. Phage was added at an MOI of 15. After 4.5 or 24 h of incubation, the samples were diluted in PBS and plated on LB plates, which were then incubated overnight at 37°C. The next day, the colonies were counted, and CFU/ml values were calculated.

This basic method was used for every liquid culture assay, with variations in media and factors added. The SDS (Sigma-Aldrich) used for the experiments depicted in Fig. S7 was prepared as a stock solution of 1 mg/ml in sterile water and diluted to experimental dilutions of 10, 100, and 200 μg/ml as needed. The stock solution was stored at 4°C and prepared fresh weekly.

### Phage spot assay.

Bacterial isolates were grown from a single colony overnight in LB at 37°C at 250 rpm. Overnight cultures were then suspended in LB top agar and placed on LB plates to solidify. ϕHP3 was diluted in LB media, and 5 μl of each dilution was dropped on the top agar. Plates were incubated overnight at 37°C. The next day, plaques were counted, and the PFU/ml values were calculated.

### Preparation of human urine.

Urine samples were collected as needed from one male and one female donor between the ages of 20 and 40. Prior to use as a media, these samples were tested for sterility by streaking on an LB plate, followed by incubation overnight at 37°C. Urine samples were kept at 4°C until use.

### Preparation of human plasma substitutes.

Human whole blood (Innovative Research, single-donor) was separated by centrifugation at 2,000 × *g* for 10 min (4°C). For heat-inactivated plasma (HIP), separated plasma was put in a water bath at 56°C for 1 h. Blood serum mimic (BSM) was prepared exactly was described by Terwilliger et al. to match human serum concentrations of sugars, salts, and amino acids (all components [pH 7.4] were purchased from Sigma-Aldrich) and sterilized using a 0.22-μm filter (36). The WBCs were then suspended in the plasma substitutes described above—or a 1:1 mix of the two—and used in the above-detailed liquid culture assay. Human serum and C3-depleted serum were purchased from Millipore Sigma. Mixtures were prepared fresh for each use. Reagents were kept at 4°C until use.

### Evolution of ϕHP3.

Bacteriophage host expansion was achieved with a patent-pending machine conceived, designed, and built in-house. The machine, comprised of linked chemostats, was designed to allow bacteriophage access to a continually refreshed source of sensitive and resistant bacteria without allowing the strains to intermix. Digital heating blocks were used to maintain temperature of culture vessels, and media flow through medical tubing was directed as shown in Fig. 6A using peristaltic pumps. Then, 500 μl of 0.22-μm filtrate and 100-kDa retentate was removed at the indicated time points and spotted onto a lawn of resistant bacteria in a typical top agar plaque assay.

### Sequencing.

Bacterial isolates were grown from a single colony overnight in LB at 37°C and 250 rpm. An E.Z.N.A. bacterial DNA kit (Omega Bio-Tek) was used according to the manufacturer’s protocol to extract genomic and plasmid DNA from the overnight cultures. Sequencing was performed by Novogene Co., Ltd. Mutations were determined using parallel methods. First, reads were trimmed, assembled, and annotated at the PATRIC Bioinformatic Resource Center using SPAdes for assembly and RAST-tk annotation (76–79). These contigs were aligned using progressiveMauve plugin for Geneious Prime 2019.2.3, and the sequence disagreements were determined manually (80). The second method used BBDuk to trim the reads and map them to a reference using the Geneious Assembler with settings to find structural variants of any size. The reference, JJ1886 (accession number CP006784.1), was chosen by determining the multilocus sequence type and *fimH* type of PATRIC-assembled contigs through the Center for Genomic Epidemiology’s MLST 2.0 and FimH Typer software (81–83). Consensus sequence of mapped contigs were then aligned using progressiveMauve and disagreements between resisters and WT strains were determined manually. Mutations highlighted here were discovered by both methods.

### Phylogenetic tree.

Assemblies from reads mapped to JJ1886 were aligned with complete genomes from representative E. coli strains using MAFFT, and these alignments were used to generate phylogenetic trees using PhyML (84–86). Branches are labeled with substitutions per site. Representative E. coli strain accession numbers were as follows: JJ1887 (CP014316), JJ2434 (CP013835), G749 (CP014488), MVAST0167 (CP014492), and K12-MG1655 (CP014225).

### Short-tail fiber structural representation.

Analysis and visualization were performed using UCSF Chimera (87). HP3 and HP3.1 short-tail fiber amino acid sequences were aligned with the amino acid sequence of ϕT4 short-tail fiber using Clustal Omega to determine the likely relative locations of the HP3 to HP3.1 mutations (88). The trimeric structure was generated by aligning the crystal structure monomer of ϕT4 short-tail fiber (PDB 1OCY) to the cryo-electron microscopy trimeric structure of the short tail fiber (PDB 1PDI; chains A to C) using UCSF Chimera’s MatchMaker. The ϕT4 residues, K473 and G474, which aligned with the mutated residues in HP3 and HP3.1, were changed to match HP3 (K464 and Y465) or HP3.1 (R464 and H465) using residues selected from the Dunbrack rotamer library (89).

### Statistics.

All statistical analyses were performed using GraphPad Prism 9.0.0. A Student *t* test or Mann-Whitney test was used as appropriate to determine the effect of phage treatment and resisters in comparison to WT in appropriate places. The log-rank (Mantel-Cox) test was used in Fig. 4B. In all cases, normality was assessed using the D’Agnostino-Pearson or Shapiro-Wilk test (*, *P* < 0.05; **, *P* < 0.01; ***, *P* < 0.01).
