# Supplementary figures and images for: Antiviral Resistance and Phage Counter Adaptation to Antibiotic-Resistant Extraintestinal Pathogenic Escherichia coli
Source: mBio. 2021 Apr 27;12(2):e00211-21. doi: 10.1128/mBio.00211-21 (PMC8092219; doi:10.1128/mBio.00211-21)

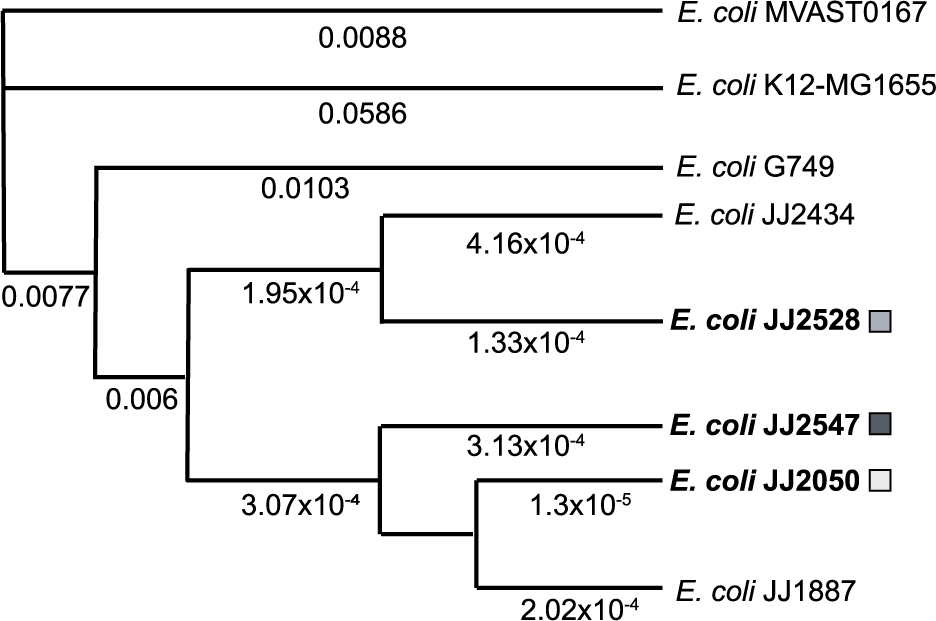

Supplement: FIG S1 [file mBio.00211-21-sf001.tif]

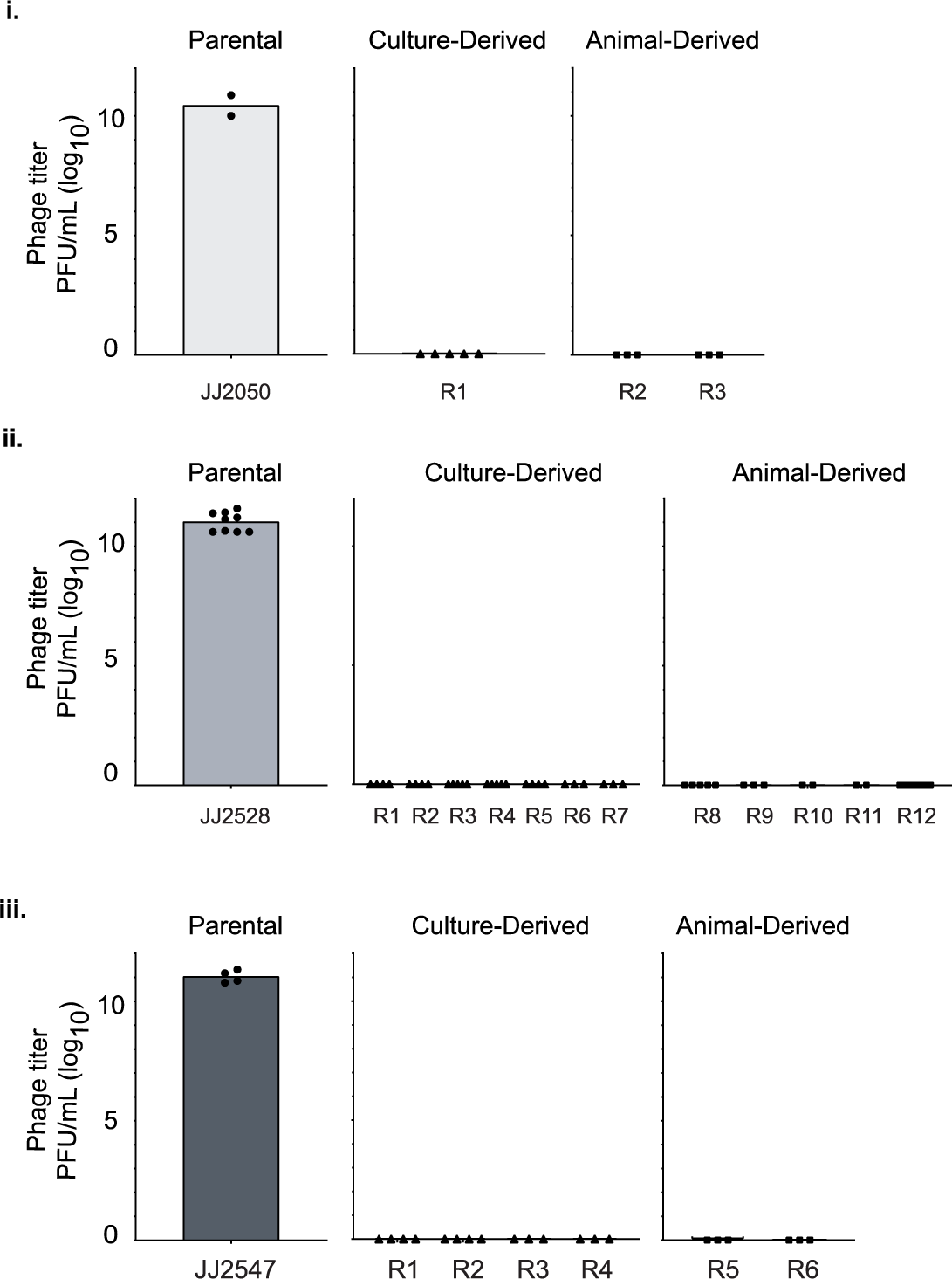

Supplement: FIG S2 [file mBio.00211-21-sf002.tif]

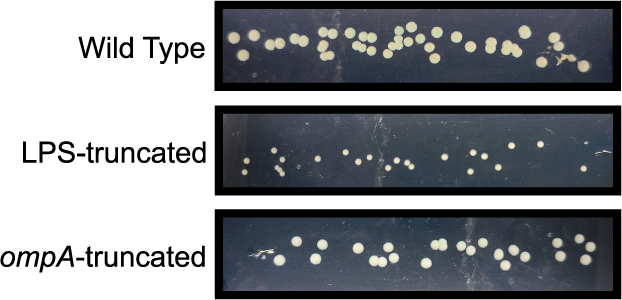

Supplement: FIG S3 [file mBio.00211-21-sf003.tif]

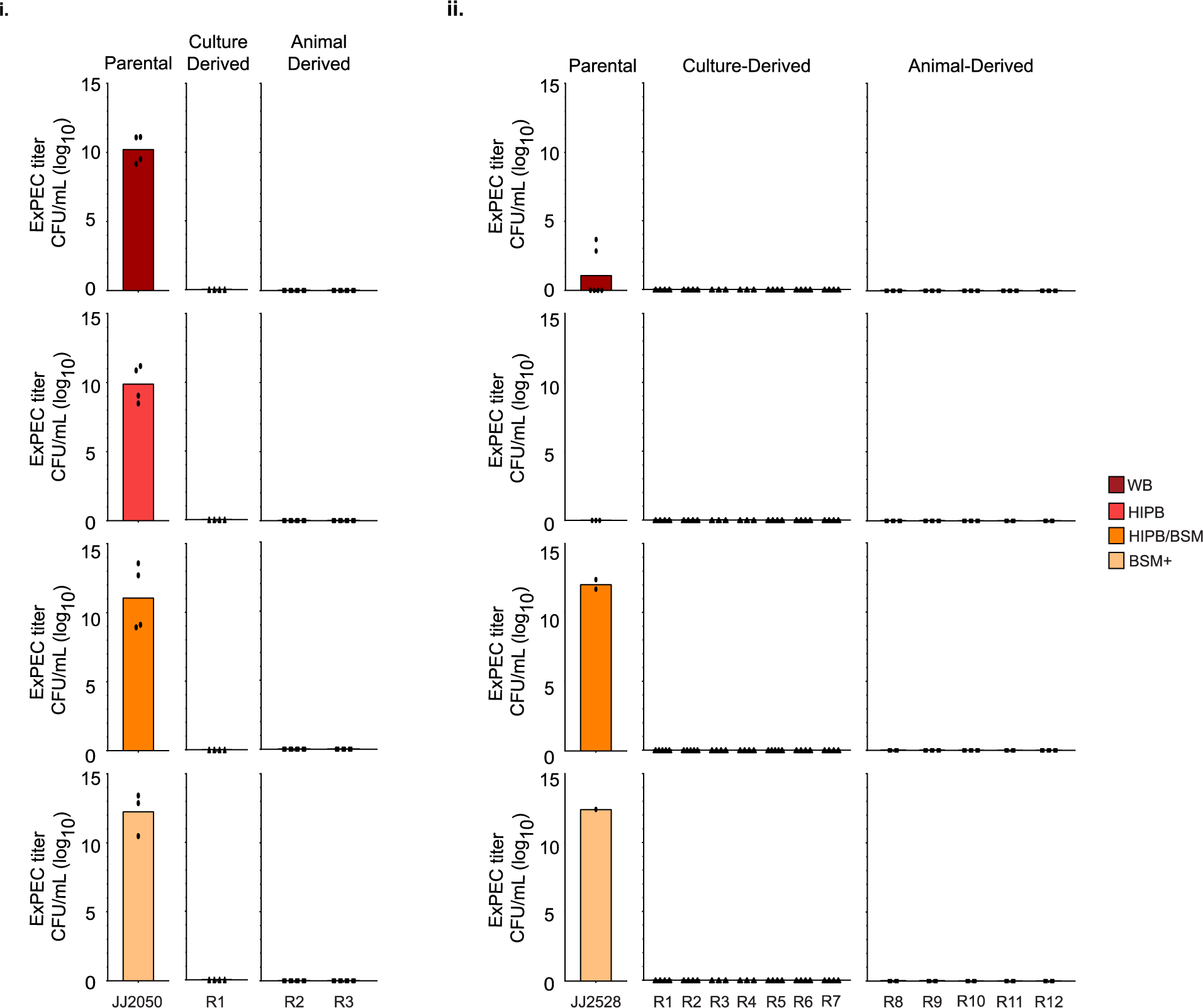

Supplement: FIG S4 [file mBio.00211-21-sf004.tif]

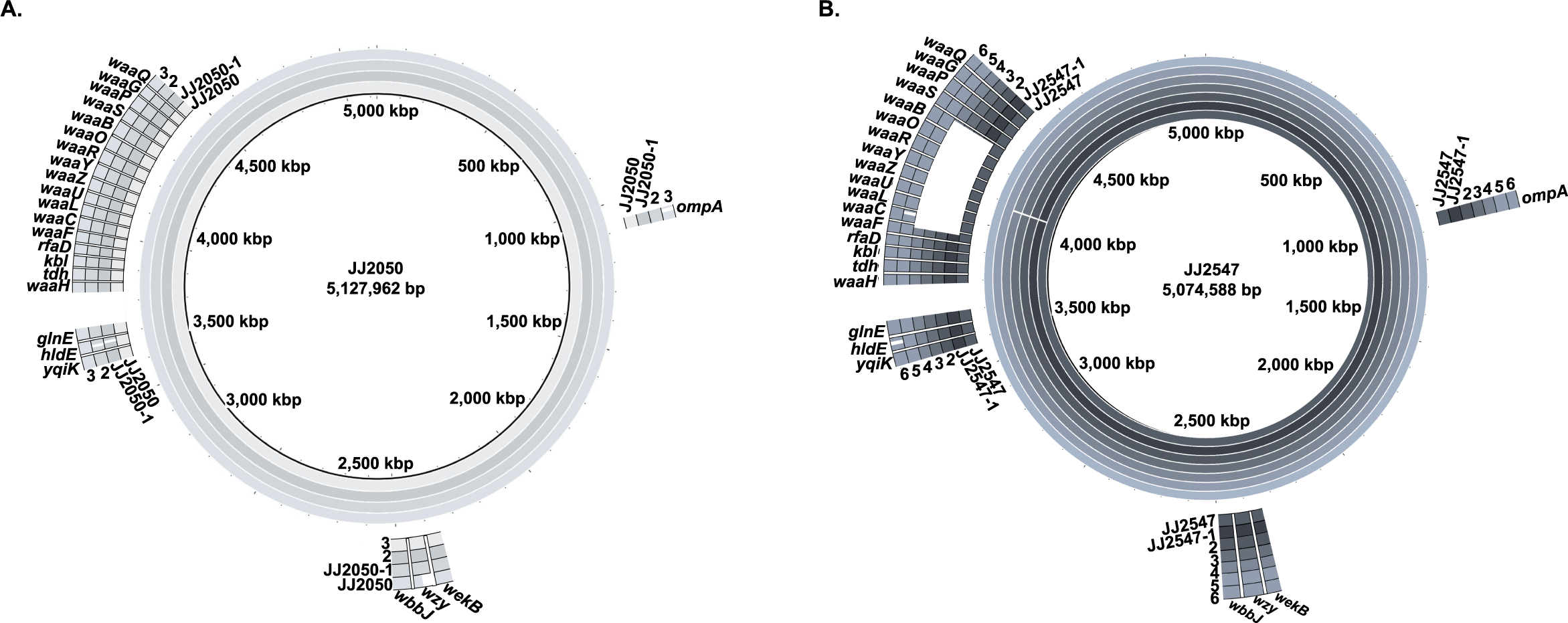

Supplement: FIG S5 [file mBio.00211-21-sf005.tif]

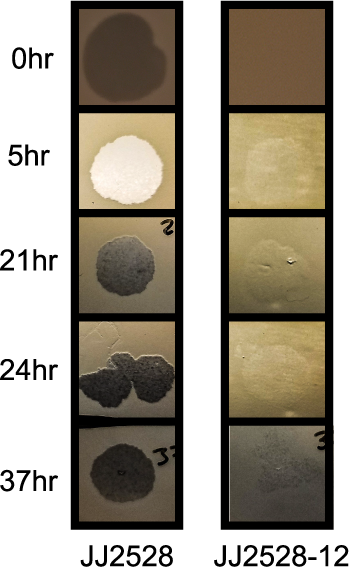

Supplement: FIG S6 [file mBio.00211-21-sf006.tif]

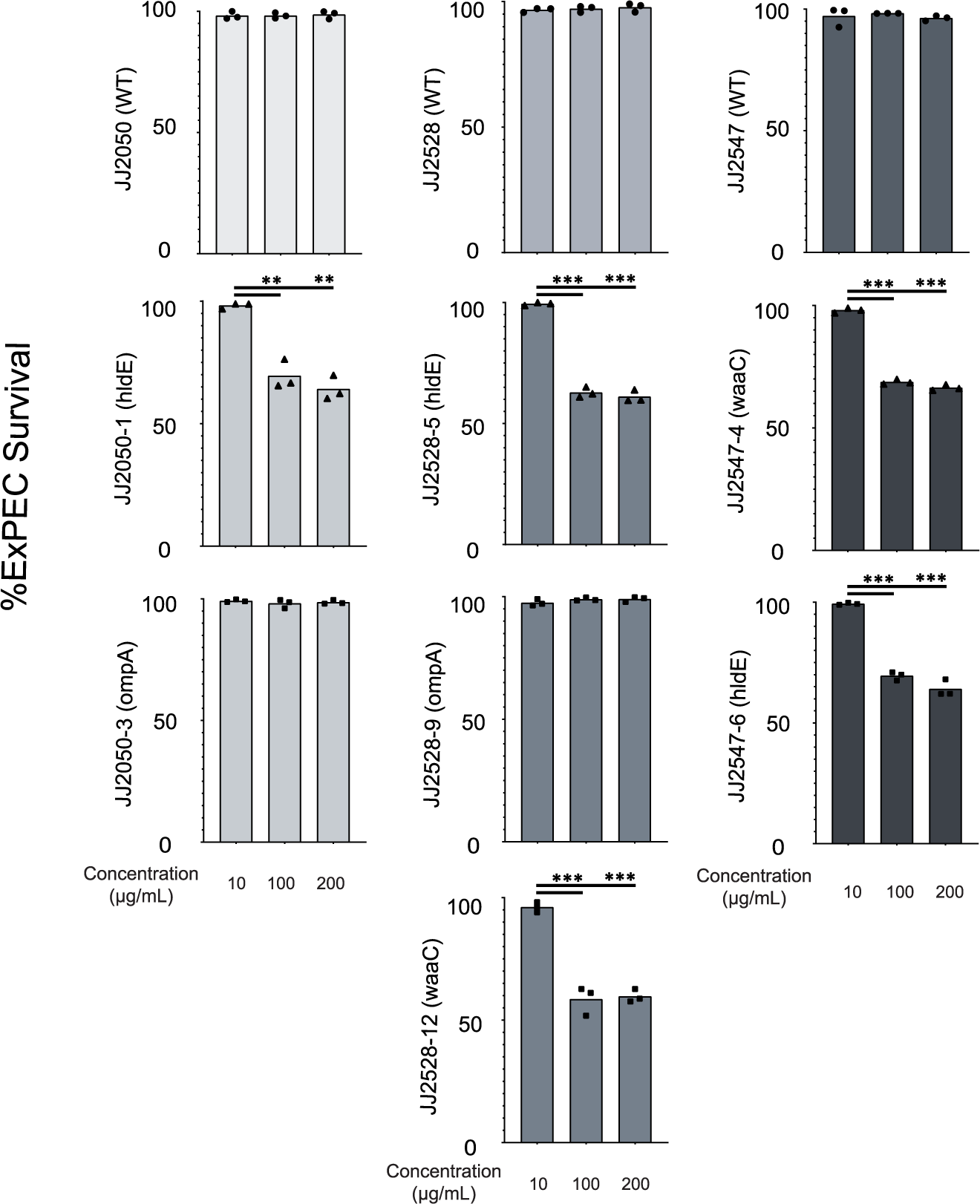

Supplement: FIG S7 [file mBio.00211-21-sf007.tif]
